# A Few-Shot Learning-Based Retinal Vessel Segmentation Method for Assisting in the Central Serous Chorioretinopathy Laser Surgery

**DOI:** 10.3389/fmed.2022.821565

**Published:** 2022-03-03

**Authors:** Jianguo Xu, Jianxin Shen, Cheng Wan, Qin Jiang, Zhipeng Yan, Weihua Yang

**Affiliations:** ^1^College of Mechanical and Electrical Engineering, Nanjing University of Aeronautics and Astronautics, Nanjing, China; ^2^College of Electronic and Information Engineering, Nanjing University of Aeronautics and Astronautics, Nanjing, China; ^3^The Affiliated Eye Hospital of Nanjing Medical University, Nanjing, China

**Keywords:** retinal vessel segmentation, few-shot learning, multi-scale class prototype, feature fusion, domain adaptability, integrated framework

## Abstract

**Background:**

The location of retinal vessels is an important prerequisite for Central Serous Chorioretinopathy (CSC) Laser Surgery, which does not only assist the ophthalmologist in marking the location of the leakage point (LP) on the fundus color image but also avoids the damage of the laser spot to the vessel tissue, as well as the low efficiency of the surgery caused by the absorption of laser energy by retinal vessels. In acquiring an excellent intra- and cross-domain adaptability, the existing deep learning (DL)-based vessel segmentation scheme must be driven by big data, which makes the densely annotated work tedious and costly.

**Methods:**

This paper aims to explore a new vessel segmentation method with a few samples and annotations to alleviate the above problems. Firstly, a key solution is presented to transform the vessel segmentation scene into the few-shot learning task, which lays a foundation for the vessel segmentation task with a few samples and annotations. Then, we improve the existing few-shot learning framework as our baseline model to adapt to the vessel segmentation scenario. Next, the baseline model is upgraded from the following three aspects: (1) A multi-scale class prototype extraction technique is designed to obtain more sufficient vessel features for better utilizing the information from the support images; (2) The multi-scale vessel features of the query images, inferred by the support image class prototype information, are gradually fused to provide more effective guidance for the vessel extraction tasks; and (3) A multi-scale attention module is proposed to promote the consideration of the global information in the upgraded model to assist vessel localization. Concurrently, the integrated framework is further conceived to appropriately alleviate the low performance of a single model in the cross-domain vessel segmentation scene, enabling to boost the domain adaptabilities of both the baseline and the upgraded models.

**Results:**

Extensive experiments showed that the upgraded operation could further improve the performance of vessel segmentation significantly. Compared with the listed methods, both the baseline and the upgraded models achieved competitive results on the three public retinal image datasets (i.e., CHASE_DB, DRIVE, and STARE). In the practical application of private CSC datasets, the integrated scheme partially enhanced the domain adaptabilities of the two proposed models.

## Introduction

Retinal vessels are important structures of our eyes, which are responsible for transporting oxygen, nutrients, and waste to ensure the normal function of the eyes. The vessels contain important information including tortuosity, diameter, angle of branches, and density, and their segmentation is crucial to the measurement of the above parameters which assist in the automatic analysis and diagnosis of various fundus diseases, such as Diabetic Retinopathy (1), Age-Related Macular Degeneration (2), and Glaucoma (3). The traditional manual segmentation method is exceedingly time-consuming and laborious, and the segmentation accuracy is easily affected by the subjective factors of doctors. Therefore, some researchers have developed segmentation methods based on traditional image processing techniques, such as the matched filtering method (4), the mathematical morphology method (5), the vessel tracking method (6), and so on. With the help of the image processing technology, the above schemes realize the transformation from manual segmentation to automatic segmentation and improve the efficiency of vessel segmentation, but there is still much room for further promotion in segmentation accuracy.

In addition, with the rapid development of artificial intelligence technology, machine learning has been widely used in various segmentation scenes (7–9) and provides a new impetus for the retinal vessel segmentation task. Various excellent machine learning-based automatic segmentation methods (10) have been designed, which can be divided into two categories (11): the unsupervised methods and the supervised methods. Among them, the unsupervised methods do not rely on pixel-wise labeling information to guide the vessel segmentation process. The typical representative is clustering. Wiharto and Suryani (12) used fuzzy c-means (FCM) algorithm to extract the vessels. They employed a channel separation, contrast limited adaptive histogram equalization (CLAHE), and median filtering to preprocess the fundus images, followed by dimension transformation, clustering, thresholding, and masking operations. The impact of the number of clusters on the segmentation effect of vessel structures was also explored in their work, which provides a direction for further improvement of the scheme. A k-means clustering-based method (13) was presented to segment the vessels, which achieved a comparable performance. The authors binarized the vessel-enhanced images, and a logical OR operation was applied on the binary vessels to produce the final results. However, it is worth noting that although the unsupervised methods do not require any label information, the single segmentation rule may lead to an unstable performance because of the differences in contrast and brightness between the retinal images.

In view of this, some ideas of vessel segmentation based on supervised learning have been proposed (14–29) and the related research work will be briefly introduced in the following. An ensemble classification-based approach was presented by Fraz et al. (14). This typical solution combined the decision tree with the conventional feature extraction steps (i.e., gradient vector field, morphological transformation, line strength measures, and Gabor filter responses), which worked well on the public datasets. With the help of image preprocessing and feature selection operation, Krishna and Gnanasekaran (15) applied the modified adaboost extreme learning machine to extract the retinal vessels, and the method performed well on pathological retinal images. To enhance the local information with better discrimination for vessel and non-vessel pixels, Aslani et al. (16) incorporated a set of robust features into a hybrid feature vector for better characterizing the retinal vessels, closely followed by a random forest classifier. Orlando et al. (17) put forward a discriminatively trained and fully connected conditional random field model to tackle the thin and elongated vessel structures, which obtained better results in terms of sensitivity, f1-score, G-mean, and Matthews correlation coefficient. A novel method (18), which regarded the segmentation task as cross-modality learning, was skillfully designed. By establishing the deep neural network with strong induction ability, they achieved satisfactory results without the feature design and preprocessing. Srinidhi et al. (19) explored the visual attention mechanism to automatically capture the most discriminative features for the random forest classifier, and a significant improvement was achieved. A simple yet effective vessel segmentation method was proposed by Jebaseeli et al. (20), the highlights of which were the operation of feature generation based on the Tandem Pulse Coupled Neural Network, and the classifier called it the Deep Learning-Based Support Vector Machine. Kaur and Mittal (21) developed a generalized scheme for retinal vessel detection and obtained a good performance. This method improved the quality of the constructed vessel features through the initial segmentation and post-processing strategy, and then, the neural network-based classifier further enhanced the accuracy of vessel segmentation.

In recent years, deep learning (DL), as one of the important technologies to realize the machine learning idea, has shown great potential in the field of medical image segmentation. Liskowski and Krawiec (22) designed a DL-based model for detecting the vessel structures. The model, with or without max-pooling layers, was trained on about 4,00,000 examples that are preprocessed with global contrast normalization, zero-phase whitening, and were augmented using geometric transformations and gamma corrections, while the area under the curve (AUC) was up to 0.99. Similarly, a deeply supervised network was established by Mo and Zhang (23), and the novelty is that the multi-level hierarchical feature extraction technique and the auxiliary classifiers are integrated into the network, which enhanced its discriminative capability on the vessel and the non-vessel pixels. Jiang et al. (24) proposed a supervised method based on the fully convolutional network (FCN) and transfer learning; the accuracy of which was 1–2% higher than other related research. To further improve the segmentation performance, a scheme inspired by a dense conditional random field was presented in (25). By training the convolutional neural network (CNN) to generate discriminative features, the scheme aimed to solve the sub-optimal problem of the hand-crafted unary features in the linear models. In order to alleviate the issue of inaccurate segmentation of thin vessels caused by the highly imbalanced pixel ratio between thick and thin vessels, a novel DL-based vessel segmentation model (26) is constructed delicately. They designed a segment-level loss to emphasize more on the thickness consistency of thin vessels in the training process and combined it with the pixel-wise loss to improve the accuracy of the vessel segmentation. Filipe et al. (27) adopted a multiscale FCN framework for the vessel segmentation. In consideration of the varying width and direction of the vessel structure, the stationary wavelet transform (SWT) was introduced into the framework to sufficiently exploit the multi-scale nature of the retinal vessels, and then, the experimental results showed the effectiveness of the method. To achieve accurate and precise retinal vessel segmentation, Park et al. (28) presented a conditional generative adversarial network called M-GAN that is composed of an M-generator and an M-discriminator. With the help of the deep residual blocks and the deeper network, the framework acquired good results. For exploring the DL-based segmentation method on the other retinal imaging modalities, a framework based on the U-net shape was established to gain the vessel mask from the scanning laser opthalmoscopy retinal images and has performed well (29).

Undoubtedly, the methods based on deep learning have greatly improved the efficiency, accuracy, sensitivity, and specificity of retinal vessel segmentation, and the end-to-end training mode also accelerates the deployment of the DL-based model in practical application scenarios. Nevertheless, this kind of scheme is driven by big data to acquire excellent intra- and cross-domain adaptability, which is challenging for medical image collection. Meanwhile, to optimize the weights of the network by the loss function, the densely annotated task is essential, which is tedious and costly. Therefore, to alleviate the above issues, a few-shot learning-based method, undertaking the task of vessel segmentation with only a few annotated training images, is proposed in this paper. The main contributions of our research are as follows: (1) Firstly, a key solution is presented for transforming the vessel segmentation scene into the few-shot learning task; (2) Then, to adapt to the vessel segmentation task, we improve the existing few-shot learning framework as the baseline model for the vessel segmentation in our work; (3) Next, we upgrade the baseline model for better utilizing the information from the support images by designing a multi-scale class prototype extraction technique; (4) After that, the skip connection technique is integrated into the upgraded model to promote the gradual fusion of the multi-scale vessel features of the query images inferred by the support image class prototype information; (5) Moreover, a multi-scale attention module is built and applied to the high-level features for the upgrade model to capture the global information to assist in vessel localization; and (6) Finally, the integrated framework is further constructed to boost the performance of both the baseline and the upgraded models in the cross-domain vessel segmentation scene.

The rest of the paper is organized as follows: Section Related Work presents the related work of few-shot learning; Section The Proposed Methods describes the details of our proposed method; Section Results and Discussions shows the experiments and discussions; and Section Conclusion and Future Work comes up with conclusions.

## Related Work

### Few-Shot Learning

Few-shot learning aims to improve the network generalization ability under the condition of a few training examples. Some methods based on this learning paradigm have been explored and applied to the classification of natural and medical images. Huang et al. (30) proposed a few-shot model for fine-grained classification. The advantage of the low-rank pairwise bilinear block was that it enhanced the effective distance metric between the support and query images. Similarly, Sun et al. (31) also studied the fine-grained classification issue based on the few-shot learning. Notably, they utilized the location mechanism to discover the similar characteristics among the objects and captured the rich discriminative information with a high-order integration. Related works are also reflected in the medical disease detection. To make up for the deficiency of the DL model in predicting rare fundus diseases, Quellec et al. (32) extended the CNN model with the few-shot learning paradigm, which improved the discrimination ability on rare pathologies through an unsupervised probabilistic way. A few-shot learning-based method (33) was presented to transfer knowledge from a well-defined source domain to a target domain, the goal of which was so the CNN model could obtain new concepts and representations from a few training samples.

### Few-Shot Segmentation

In addition, the few-shot method also performs well in the natural image segmentation field. Seeing that the pixel-wise segmentation is tedious and costly, a segmentation network (34), consisting of a two-branch dense comparison module and an iterative optimization module, which is followed by an attention block, was proposed and has achieved better performance. Li et al. (35) exploited a similar technique that integrated the attention mechanism and the refinement network into the segmentation model, which improved the model performance. Compared with the natural images, the task of the medical image segmentation is more complex and difficult due to the similarity between the normal tissues and the pathological regions, and the extreme professionalism of the pixel-wise annotation process. Some of the few-shot segmentation methods have been successfully applied to the medical image segmentation, which affords new ideas for solving the medical image segmentation problem. Feyjie et al. (36) designed a few-shot learning-based framework for the skin lesion segmentation, the excellent performance of which also provided inspiration for our research work. To enhance its segmentation ability, they also incorporated the semi-supervised block into the framework. Besides, a unified framework (37), which worked under the condition of the scarcity of both the medical images and the corresponding annotations, was put forward and has contributed to the rare disease segmentation. Additionally, Ouyang et al. (38) further developed creatively a few-shot-based method without any annotations and trained the network with only the pseudo labels, which opened up a new direction for few-shot-based segmentation schemes.

It can be found that the few-shot learning has been widely used in the field of natural image classification and segmentation, which promotes the paradigm to show its head in the similar fields of medical images. Also, its successful application in practice drives us to further apply it in the retinal vessel segmentation to assist in fundus CSC laser surgery. The motivation of introducing the few-shot learning into the fundus blood vessel segmentation task is that the paradigm can guide the vessel segmentation model training under the condition of a small number of annotated images, which is different from most machine learning models that rely on a large amount of image resources for an effective feature learning. This does not only reduce the cost of data collection but also helps ease the pressure of label making. However, we are faced with a thorny problem that has to be solved, that is, how to transform the vessel segmentation scene into a few-shot segmentation task? Specifically, how to construct the support and query sets in our vessel segmentation task? The solution will be introduced in Section The Proposed Methods.

## The Proposed Methods

### Methods

**Figure 1:**
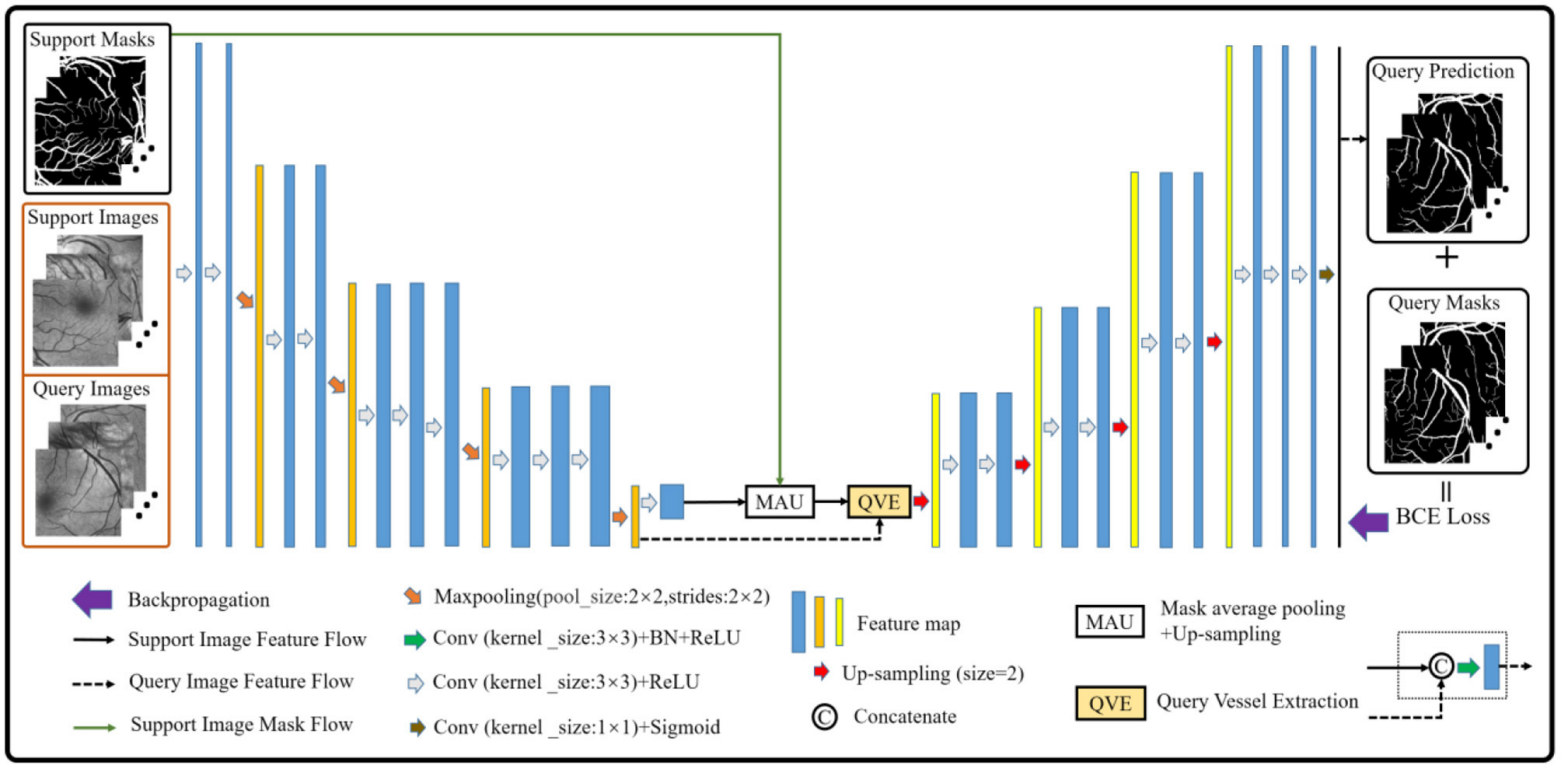
An overview of the baseline model.

The vessel segmentation scheme based on the few-shot learning paradigm consists of two parts. The first part is how to construct the support and the query sets in vessel segmentation task by imitating the natural image scene. As we all know, the vessel structure of each person is not exactly the same. Therefore, in this paper, the vessel images from different people are regarded as different classes, and the patches of each class are regarded as its members. Then, we sampled the members of different classes as the support set, and the remaining members of the corresponding classes as the query set. Especially, given a C-way K-shot learning task, this simple yet effective solution can smoothly build the corresponding episodes for the vessel segmentation task. The second part is to establish the vessel segmentation model. Inspired by (36), we build a model in which the semi-supervised module in (39) is removed to suit our task. Simultaneously, the dilated convolution kernels are also replaced by the ordinary convolution kernels to prevent information loss caused by the gridding effect. Finally, we obtain the baseline model for retinal vessel segmentation as shown in Figure 1.

### Preprocessing

**Figure 2:**
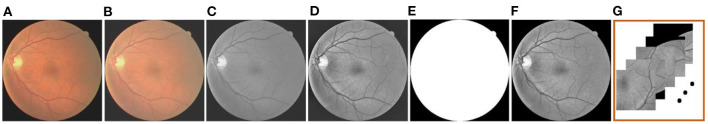
The preprocessing operation: **(A)** Original image; **(B)** Gamma-corrected image; **(C)** Green channel image; **(D)** CLAHE image; **(E)** Mask; **(F)** Mask-processed image; and **(G)** Patches.

After solving the problem that transformed the vessel segmentation scene into a few-shot segmentation task, here comes the preprocessing operation. In this paper, the gamma correction and the CLAHE are employed, respectively, aiming at improving the brightness and the contrast of the retinal images. In addition, we separate the green channel, which is a routine operation in the vessel segmentation scenario, to show the vessel structures more clearly. The masks are used to eliminate the disturbance outside the region of interest. Furthermore, in response to the construction of the support and the query sets, the clipping operation is applied to the previous operation result. Meanwhile, the overlapping technique is also adopted here to ensure the similarity and the quantity of patches in the same retinal image. Specifically, we firstly set the size of the image patch template to 224 × 224 pixels, and then the clipping function is realized by moving the template in the horizontal and vertical directions. In the process, we realize the overlapping function by setting the moving step as 64 (i.e., the overlapping size is 160) among each image patches in the above directions. The whole preprocessing is shown in Figure 2.

### Problem Definition

The task of this paper is to construct the vessel segmentation model based on the few-shot learning paradigm, which possesses a strong generalization ability to the similar unseen targets by learning only a few annotated examples. In this scenario, we are given an annotated retinal image set $D={\left\{X_{i},Y_{i}\right\}}_{i=1}^{N}$, where *X*_*i*_ is the *i*^*th*^ sample and *Y*_*i*_ corresponds to its label. The N is the number of the annotated retinal images. In order to train the model, D is divided into the training set $D_{train}={\left\{X_{j},Y_{j}\right\}}_{j=1}^{N_{1}}$, validation set $D_{valid}={\left\{X_{k},Y_{k}\right\}}_{k=1}^{N_{2}}$, and testing set $D_{test}={\left\{X_{l},Y_{l}\right\}}_{l=1}^{N_{3}}$, where *N1, N2*, and *N3* represent the number of images in each set, respectively. As mentioned above, in order to adapt to the few-shot segmentation task, each retina image *X*_*i*_ is regarded as a unique class and is cropped into patches to build the support set $S_{is}={\left\{{x_{i}}_{s},y_{is}\right\}}_{s=1}^{K}$ and the query set ${Q_{i}}_{q}=\left\{{x_{i}}_{q},y_{iq}\right\}$, where *K* denotes the sampling numbers from the class *i*, _*x*_*i*_*s*_ is the *s*^*th*^ support patch extracted from the image *X*_*i*_ with its annotation *y*_*is*_, and _*x*_*i*_*q*_ is the query patch extracted from the remaining patches in the image *X*_*i*_ with its annotation *y*_*iq*_. So, given a *C*-way *K*-shot segmentation task, the training episodes can be described as $\left(S_{js},{Q_{j}}_{q}\right)$ by randomly extracting *C* classes from *N1* and *K* members from each corresponding class during the training stage. To verify the model performance in the training process and to save the optimal weight parameters, the same technique, such as building the training episodes, is also applied to the validation set $D_{valid}$.

### Few-Shot Learning-Based Segmentation Architecture

**Figure 3:**
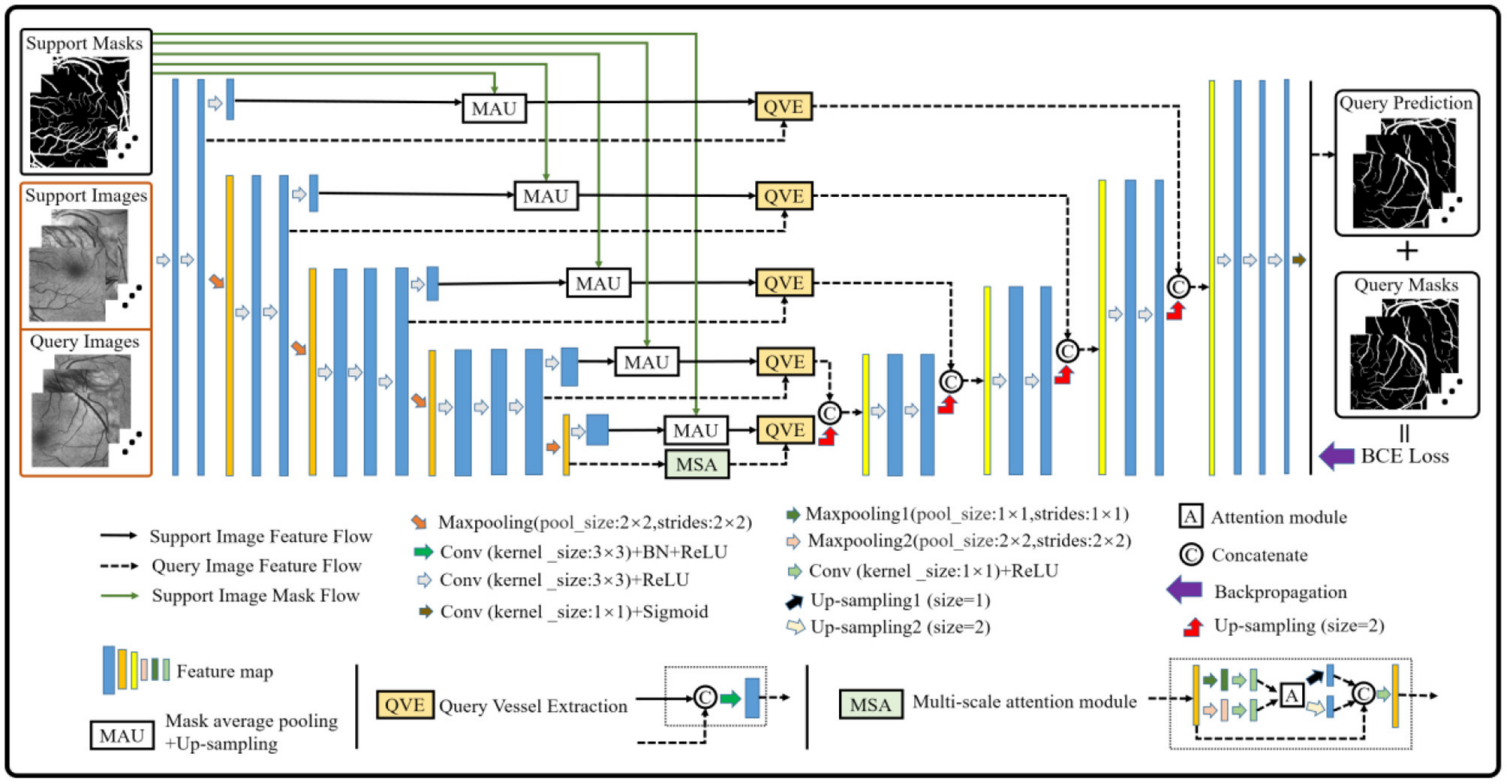
An overview of the upgraded model.

Although the baseline model performs well in the vessel segmentation task (refer to Section Results and Discussions), it is defective to derive the label information of the query images by only using the support image class prototype information from the high-level layer. The reason is that part of the foreground and the background contents of the support images will be lost in the down-sampling process, which will provide an incomplete guidance for the query image label prediction and render inaccurate results. Therefore, the baseline model is further upgraded to alleviate the above problems (as shown in Figure 3). Specifically, a multi-scale class prototype extraction technique, which is embedded into the parallel positions of the down-sampling steps in the encoder composed of the first four modules from the VGG16 (40), is designed for the information derivation of the query images. Besides, the gradual fusion scheme for the multi-scale vessel features is also integrated in the upgraded model. In this paper, the mask average pooling (36, 38) is also employed to extract the class prototype information of the support images, and the formulation can be expressed as:


(1) $$\begin{array}{l} P_{s}^{l}=\frac{1}{W\times H}\overset{W}{\underset{w=1}{\sum }}\overset{H}{\underset{h=1}{\sum }}f_{\omega }\left(x_{s}^{l}\right)◦y_{s}^{l},l\in \left(1,2,3,4,5\right) \end{array}$$

where $P_{s}^{l}$ represents the class prototype of the support image feature map $x_{s}^{l}$ with the width *W* and height *H* and *l* is the parallel position mark. The *f*_ω_ is the feature mapping function composed of weight parameters ω and the network framework, and $y_{s}^{l}$ denotes the annotation obtained by the linear interpolation sampling on the support image mask. The Hadamard product, denoted by the symbol ° , is used to extract the class prototype of $x_{s}^{l}$. Then, the same operation is adopted as (29), that is, to apply the up-sampling operation on each class prototype $P_{s}^{l}$ and convolve the sampling result with the corresponding query image feature map $x_{q}^{l}$ to obtain the multi-scale vessel maps. Meanwhile, the skip connection technique is integrated into the upgraded model to fuse the multi-scale information for better segmentation results. Finally, the binary cross-entropy loss function is established based on the probability prediction map $p_{jk}^{q}$ and its corresponding true annotation *y*_*jk*_, which can be expressed as:


(2) $$\begin{array}{l} L\left(\omega ,b\right)=-\frac{1}{C\times K}\overset{C}{\underset{j=1}{\sum }}\overset{K}{\underset{k=1}{\sum }}[y_{jk}\cdot log\left(p_{jk}^{q}\right)\\ \;+\left(1-y_{jk}\right)\cdot log\left(1-p_{jk}^{q}\right)] \end{array}$$

(3) $$\begin{array}{l} p_{jk}^{q}=sigmoid\left(x_{Q}\left(k\right)\right)=\frac{1}{1+e^{-x_{Q}\left(k\right)}} \end{array}$$

where $p_{jk}^{q}$ and *x*_*Q*_(*k*) denote the probability prediction map of the query patches with its true annotation, and the *k*^*th*^ feature map of the query patches of the model outputs, respectively. In addition, it is well-known that the convolution module is a local operation and ignores the global information, which may lead to the failure of a pixel-level prediction task. Considering this, a block (i.e., the MSA module in Figure 3) based on the non-local attention operation (41) and multi-scale operation is established to seek the global information integration of the query feature maps at different scales, aiming at assisting the upgraded model to approach the retinal vessel segmentation task. In view of the rich semantic information of high-level features and the cost of mathematical calculation, the attention operation is just performed on the query feature map with two scales at the last layer of the encoder, and the details are shown in Figure 3.

**Figure 4:**
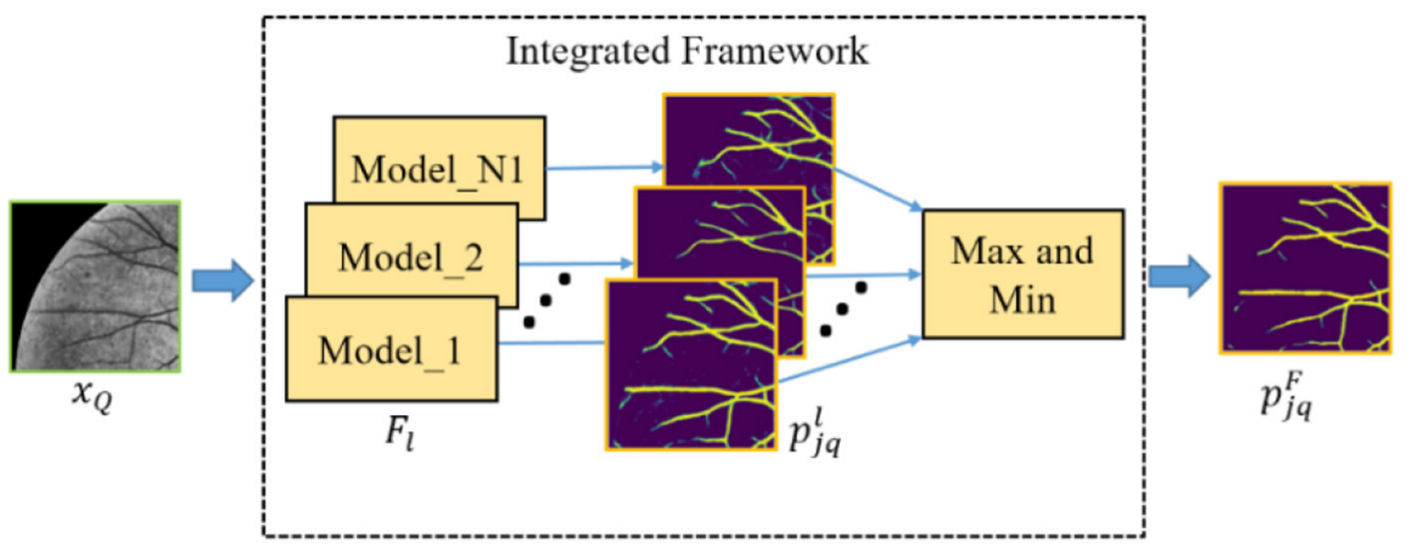
An overview of the integrated framework.

Furthermore, in order to obtain the good performance of both the baseline and upgraded models in the cross-domain retinal vessel segmentation task, an integrated framework (as shown in Figure 4) is conceived according to the principle that the minority obeys the majority, the mathematical idea of which can be parameterized as follows:


(4) $$\begin{array}{l} p_{jk}^{l}=F_{l}\left(x_{Q}\left(k\right)\right),\;l=1,...,N_{1} \end{array}$$

(5) $$\begin{array}{l} p_{N1}=1\left\{\left(\overset{N_{1}}{\underset{l=1}{\sum }}1\left\{p_{jk}^{l}>T\right\}\right)>T_{1}\right\} \end{array}$$

where *N*_1_ and *F*_*l*_are the total number of models in the integrated framework and the *l*^*th*^ model, respectively. The $p_{jk}^{l}$and *p*_*N*1_ denote the probability prediction map from the *l*^*th*^ model and the statistical prediction result of *N*_1_ models, respectively. The 1{•} is an indicator function which outputs 1 if the parameters meet the threshold condition *T* or *T*_1_, and these two values are set to 0.5 and 1 in this paper, respectively. Then, the final probability prediction map $p_{jk}^{F}$ of the query patches can be expressed as:


(6) $$\begin{array}{l} p_{jk}^{F}=\{\begin{array}{c} \max\left\{p_{jk}^{l}\left(m,n\right)\right\},\;if\;p_{N_{1}}(\text{m},\text{n})=1\\ \min\left\{p_{jk}^{l}\left(m,n\right)\right\},\;otherwise\; \end{array} \end{array}$$

where (*m*, *n*) represents the position coordinate of the element in $p_{jk}^{l}$ or *p*_*N*1_.

### Segmentation Architecture Configuration

In this paper, no matter our baseline model or upgraded model, the basic architecture configuration is the same, which is composed of encoder and decoder. The encoder consists of the first four modules of VGG16 (40), and the decoder consists of conventional neural network layer. Table 1 shows the specific configuration.

**Table 1:** The basic configuration of the segmentation architecture.

**Encoder**	**Decoder**
Conv3-64	Up-sampling
Conv3-64	Conv3-512
Maxpooling	Conv3-256
Conv3-128	Up-sampling
Conv3-128	Conv3-256
Maxpooling	Conv3-128
Conv3-256	Up-sampling
Conv3-256	Conv3-128
Conv3-256	Conv3-64
Maxpooling	Up-sampling
Conv3-512	Conv3-64
Conv3-512	Conv3-32
Conv3-512	Conv3-3
Maxpooling	Conv3-1

## Results and Discussions

### Retinal Image Datasets and Experimental Settings

To assess the performance of the proposed baseline and upgraded models based on the few-shot learning paradigm, we have carried out extensive experiments on the public retinal image datasets, namely, CHASE_DB (42) (abbreviated as CHASEDB), DRIVE (43), and STARE (44), to demonstrate the excellent potential of the paradigm. In our experiments, for each dataset, only 10 images, namely, 10 classes, are used as the candidate set for constructing episodes composed of the support and query sets, which is different from the machine-learning-based segmentation models that are generally trained with more than 10 images. In fact, we randomly selected no more than five types of vessel images, that is, *C* is set to 5, 4, or 3, to drive the vessel segmentation model in the training process. The remaining images are divided into the validation and testing sets. Of note, in order to deduce the label information of the testing set based on the structural similarity between retinal vessels, the testing images share the support images of the validation set. In addition, taking the calculation cost and the reliability verification of both the baseline and the upgraded models into consideration, five groups of *C*-way *K*-shot modes (Hereinafter referred to as *CK* modes), namely, 5-way 3-shot, 4-way 3-shot, 3-way 3-shot, 3-way 4-shot, and 3-way 5-shot, are set up to construct different episodes for training the above vessel segmentation models, respectively. In addition, we conducted application experiments on ophthalmic clinical CSC dataset composed of 20 fundus images to test the effectiveness of the two models and the feasibility of the integrated framework. All experiments are performed on the NVIDIA-3080ti GPU with the Tensorflow framework, and the learning rate, epoch, iteration, and gradient optimizer are set to 0.0001, 30,100, and Adam, respectively.

### Evaluation Metrics

In this section, the performance of the baseline and upgraded models in the training, validation, and testing processes is comprehensively evaluated based on the accuracy and the loss of training process, the segmentation metrics, namely, sensitivity (Sen), specificity (Spe), accuracy (Acc), f1-score (F1), and AUC, and the actual segmentation results on the testing images. Meanwhile, the superiority and the potential of the proposed method is also discussed by comparing it with some typical machine learning based vessel segmentation methods. The segmentation metrics mentioned above are written as:


(7) $$\begin{array}{l} sensitivity=tp/(tp+fn),specificity=tn/(tn+fp) \end{array}$$

(8) $$\begin{array}{l} accuracy=\left(tp+tn\right)/(tp+fn+tn+fp),\\ f1-score=2tp/(2tp+fn+fp) \end{array}$$

where *tp, tn, fn*, and *fp* denote true positive, true negative, false negative, and false positive, respectively.

### Comparison of the Baseline and Upgraded Models on the Training and Validation Sets

**Figure 5:**
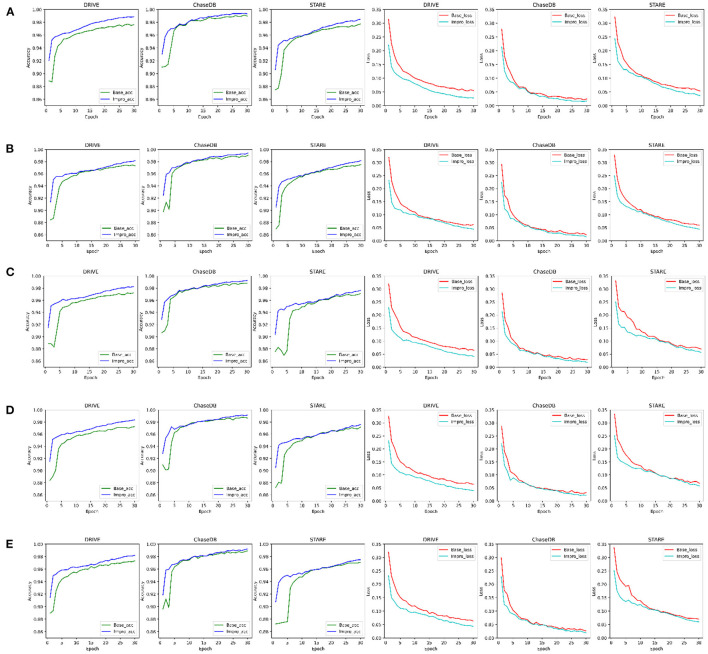
Comparison of the baseline and upgraded models on training set: **(A)** 5-way 3-shot; **(B)** 4-way 3-shot; **(C)** 3-way 3-shot; **(D)** 3-way 4-shot; and **(E)** 3-way 5-shot.

For convenience, in the following sections, the baseline and upgraded models are represented by “Base” and “Impro”, respectively. As shown in Figure 5, it can be found that both models can achieve more than 95% accuracy in the end. The segmentation accuracy of the Impro model is higher than that of the Base model in most cases under different *CK* modes on three datasets during the training process. Similarly, compared with the Base model, the loss value of the Impro model keeps a low state on the whole. Even in some training epochs, the difference of this loss value between the two models is relatively small, but it is maintained for a short time. In addition, it can also be found that the upgraded model shows the advantages in the initial stage of training, whether it is the accuracy value or the loss value. Specifically, at the beginning, the initial accuracy of the upgraded model is higher than that of the baseline model, and the loss value is also small, which means that the upgraded operation can effectively improve the learning ability of the model and promote the model to quickly capture the vessel and the non-vessel information in the fundus images. In general, the performance of the upgraded model is slightly superior to that of the baseline model in terms of accuracy and loss on the training set.

**Figure 6:**
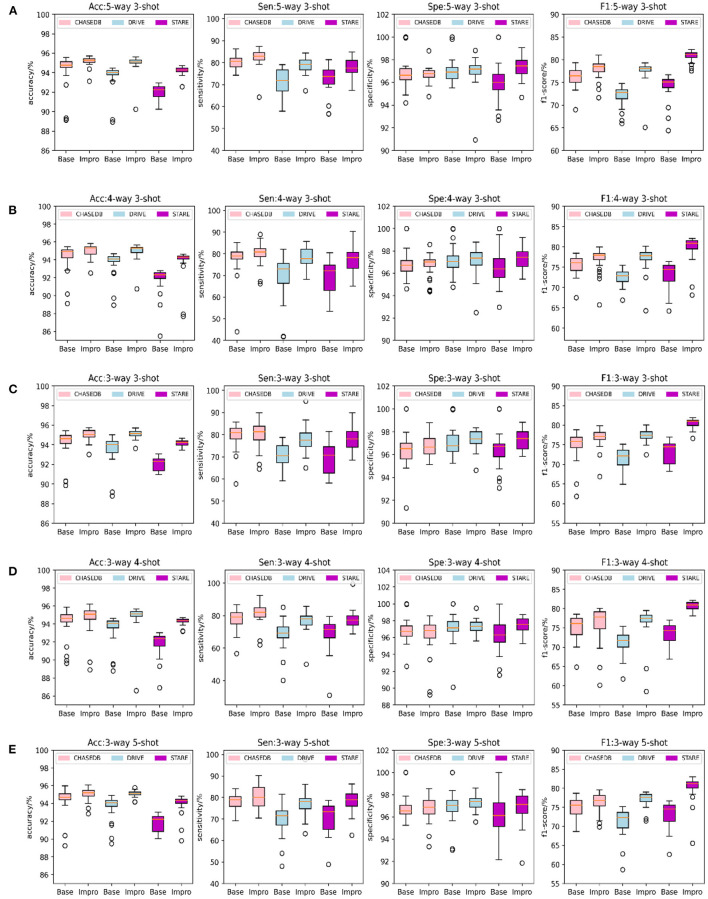
Comparison of the baseline and upgraded models on validation set: **(A)** 5-way 3-shot; **(B)** 4-way 3-shot; **(C)** 3-way 3-shot; **(D)** 3-way 4-shot; and **(E)** 3-way 5-shot.

Furthermore, to check the generalization ability of the model to the unseen vessel images after each epoch and to save the optimal weights in time, the accuracy, sensitivity, specificity, and f1-scores of the two models on the validation set are specially counted and plotted as shown in Figure 6. It shows that the Impro model surpasses the Base model in terms of the average segmentation accuracy and average f1-score in different *CK* modes from Figure 6, which implies that the upgraded model has a better segmentation performance. When referring to the other two metrics (i.e., average sensitivity and average specificity), we can also get the conclusion that the segmentation performance of the baseline model is inferior to that of the upgraded model in most cases. Moreover, the experiment can also show that there are some differences in the performance of either the baseline model or the upgraded model on different datasets, which is mainly caused by the inconsistent data distribution of each datasets. Nevertheless, compared with the baseline model, the average values of various metrics of the upgraded model are relatively consistent on different datasets, which reflects its strong learning ability. However, it is worth noting that although the Impro model performs well on the whole, its robustness still needs to be enhanced.

### Comparison of the Baseline and Upgraded Models on the Testing Set

**Figure 7:**
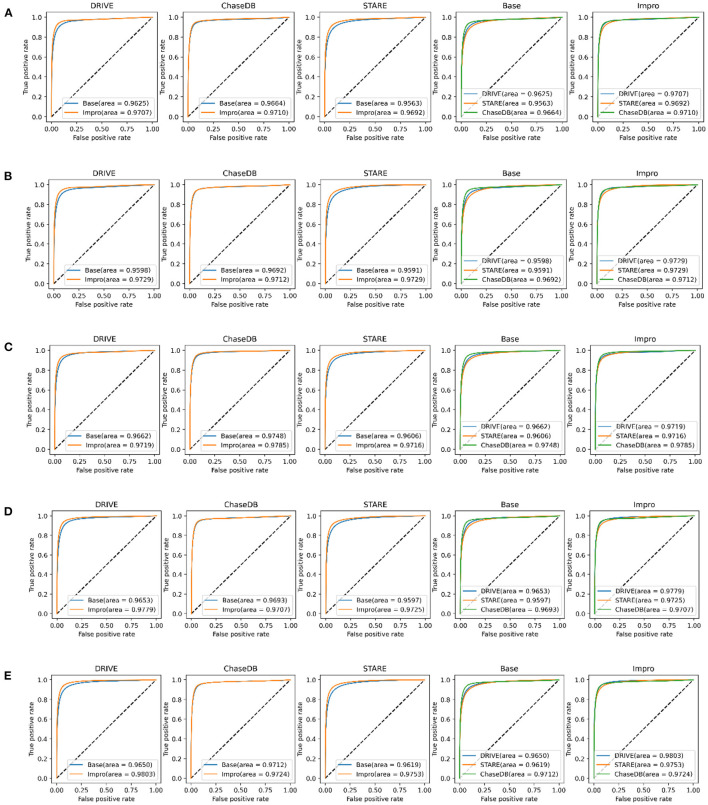
Comparison of the baseline and upgraded models based on the AUC: **(A)** 5-way 3-shot; **(B)** 4-way 3-shot; **(C)** 3-way 3-shot; **(D)** 3-way 4-shot; and **(E)** 3-way 5-shot.

It can be obviously revealed from Figures 7A–E that both models can achieve higher AUC values. Especially on the DRIVE dataset, the AUC value of the Impro model is as high as 98.03% under the combination of 3-way 5-shot mode and is better than most of the listed methods. Under different *CK* modes, the AUC values of the Impro model on the DRIVE, CHASEDB, and STARE datasets are higher than those of the Base model, which not only explicitly proves the excellent performance of the upgraded model, but also shows the necessity and the effectiveness of the upgraded operation. Moreover, from Figures 7A–E, we can directly see that the AUC values of the Base model or of the Impro model are different in each dataset, which indicates that the dataset, itself, is an import factor affecting the model performance, and also proves once again that the inconsistency of data distribution is a significant part leading to a difference of the model performance.

**Figure 8:**
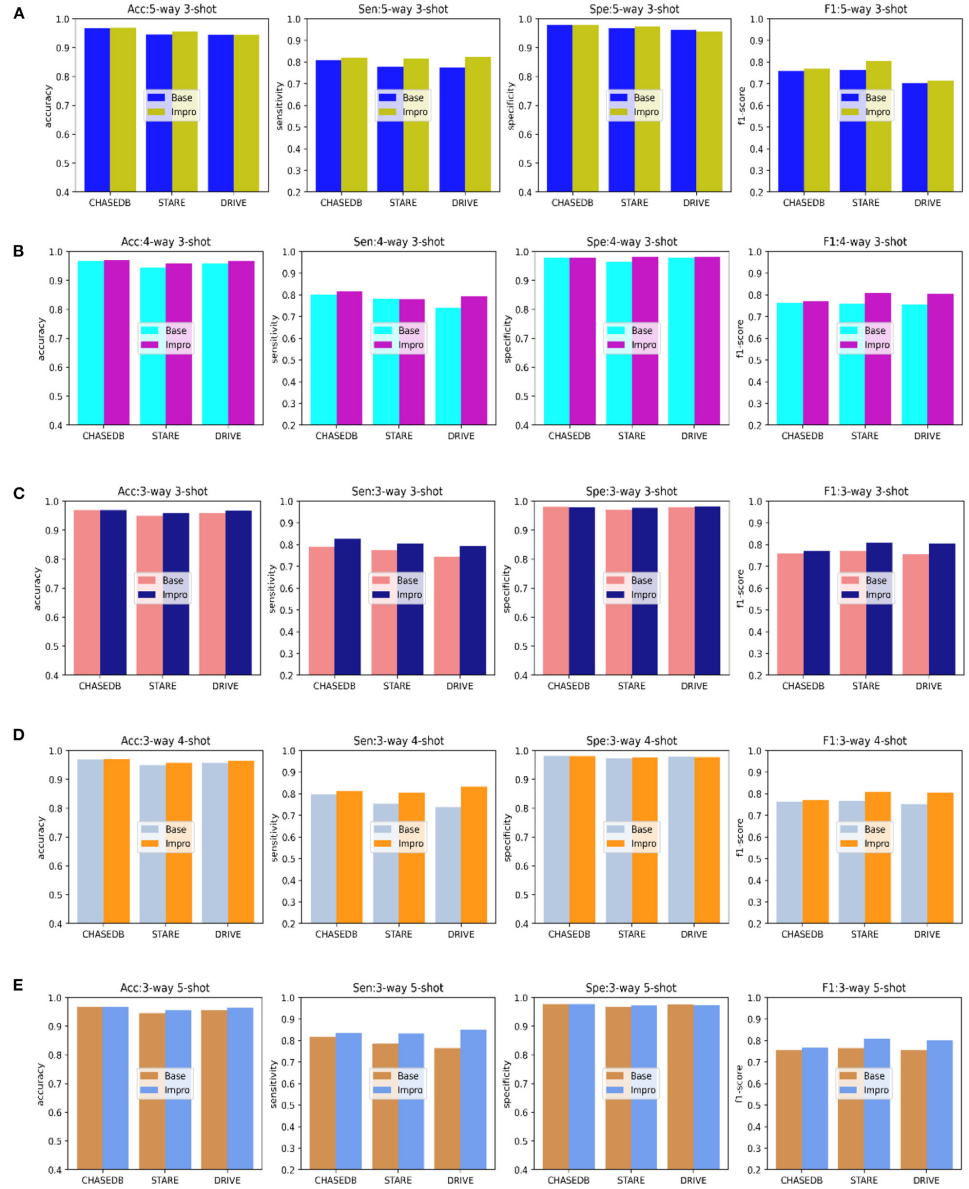
Comparison of the baseline and upgraded models based on the sensitivity, specificity, accuracy, and f1-score: **(A)** 5-way 3-shot; **(B)** 4-way 3-; **(C)** 3-way 3-shot; **(D)** 3-way 4-shot; and **(E)** 3-way 5-shot.

In addition to the AUC value, we also evaluate the generalization ability of the models on the testing set from the other four evaluation metrics shown in Figure 8. Under different *CK* modes, both the two models achieve more than 90% in terms of the accuracy and specificity values, and overall, the Impro model surpasses the Base model on the above values. Besides, in consideration of the sensitivity and the f1-score values, the conclusion can be drawn that the Impro model is significantly better than those of the baseline model in most cases on the three testing sets, which is similar to their performance on the validation set. In general, the performance of the two models in the testing sets is consistent with that in the training and validation sets, which implies that both the two models can better transfer the knowledge learned from the training set to the unseen vessel images. However, it can still be found that the test results of the two models in different datasets are slightly different, which is also consistent with the previous experimental observations. The difference in the model performance caused by the inconsistent data distribution of different datasets is the common factor of almost all the current vessel detection methods based on machine learning, and it is also a problem to be alleviated in the future.

### Comparison of the Baseline and Upgraded Models on the Segmentation Results

**Figure 9:**
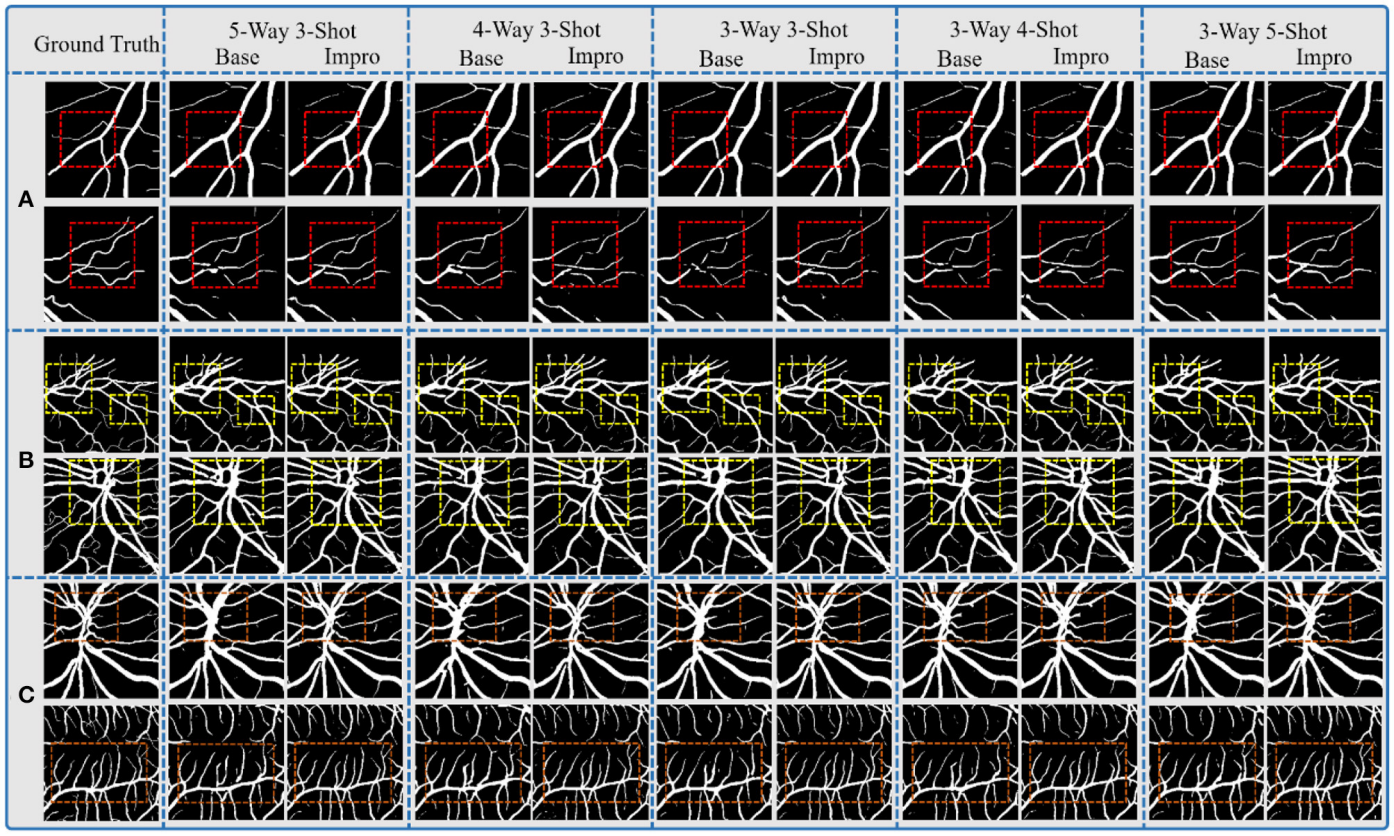
Comparison of the baseline and upgraded models on segmentation results: **(A)** CHASEDB; **(B)** DRIVE; and **(C)** STARE.

Through the above analysis, on the one hand, it shows the excellent performance of both the Base and the Impro models in the vessel segmentation task, while on the other hand, the Impro model outperforms the Base model overall, which proves the necessity for the upgraded operation. Furthermore, Figure 9 shows the details of the two models in the vessel segmentation task on three datasets. It can be found that the two models have good segmentation ability for the wide vessels and can extract the main vessel structures. At the same time, by observing the overall segmentation performance of the two models on the three datasets, compared with the Base model, the Impro model, still, has the upper hand, which is embodied in the vessel continuity and adhesion. Specifically, it can be clearly seen from Figure 9B that the wide vessels segmented from the Base model may adhere to each other, but the Impro model performs better and can properly deal with the vessel adhesion events. Meanwhile, the Base model is generally inferior to the Impro model in terms of the vessel continuity. For instance, as shown in Figure 9C, there is a certain degree of discontinuity between the branch vessel located by the baseline model and the main vessel located by the same model. However, compared with this, the continuity between the main and the branch vessels detected by the upgraded model is relatively satisfactory.

However, although the Impro model has made progress in the above segmentation performance, it cannot be ignored that the baseline and upgraded models still have shortcomings in small vessel segmentation, and this defect is also a common weakness of the existing vessel segmentation methods. The upgraded operation, indeed, improves the recognition ability of the Base model for small vessels, but there is still much room for improvement, which is also the direction of our future efforts.

### Comparison of the Baseline and the Upgraded Models With the Previous Studies

In this section, we compare the proposed baseline and upgraded models with the existing typical segmentation schemes based on machine learning, and the results for each dataset are summarized in Tables 2–**4**.

**Table 2:** Performance comparison on DRIVE.

**Methods**		**Acc (%)**		**Spe (%)**		**Sen (%)**		**AUC (%)**	
		**Base**	**Impro**	**Base**	**Impro**	**Base**	**Impro**	**Base**	**Impro**
Proposed	5-way 3-shot	94.52	94.46	96.08	95.56	77.48	82.45	96.25	97.07
	4-way 3-shot	95.82	96.61	97.91	98.28	73.97	79.21	95.98	97.29
	3-way 3-shot	95.82	**96.64**	97.88	**98.29**	74.35	79.47	96.62	97.19
	3-way 4-shot	95.77	96.45	97.87	97.70	73.90	83.35	96.53	97.79
	3-way 5-shot	95.66	96.34	97.50	97.43	76.55	**84.95**	96.50	**98.03**
Machine learning	Fraz et al. (14)	94.80		98.07		74.06		97.47	
	Krishna et al. (15)	96.19		**98.36**		74.35		-	
	Aslani and Sarnel (16)	95.13		98.01		75.45		96.82	
	Orlando et al. (17)	-		96.84		78.97		95.07	
	Li et al. (18)	95.27		98.16		75.69		97.38	
	Srinidhi et al. (19)	95.89		96.67		**86.44**		97.01	
	Liskowski and Krawiec (22)	95.35		98.07		78.11		97.90	
	Mo and Zhang (23)	95.21		97.80		77.79		97.82	
	Jiang et al. (24)	96.24		98.25		75.40		98.10	
	Zhou et al. (25)	94.69		96.74		80.78		-	
	Yan et al. (26)	95.42		98.18		76.53		97.52	
	Filipe et al. (27)	95.76		98.04		80.39		98.21	
	Park et al. (28)	**97.06**		**98.36**		83.46		**98.68**	

*The bold values indicate the maximum value of the corresponding metric in the manuscript*.

For the DRIVE dataset, it can be found that the accuracy, specificity, sensitivity, and AUC values of the Impro model all exceeded the Base model under different *CK* modes, and the sensitivity difference between the two models is the most obvious, which proves that the upgraded model can better distinguish the vessel pixels from the background pixels. Besides, the highest accuracy, specificity, and sensitivity of the Impro model are 96.64, 98.29, and 84.95%, respectively, which are not only higher than those of the Base model but are also significantly competitive compared with most of the schemes listed. In addition, the AUC value of the upgraded model is also visibly superior to the baseline model, and the biggest difference is 1.53%. Moreover, the Impro model is just 0.65% smaller in this metric than the optimal one that is listed, which not only reveals its potential superiority but also means that there is still room for improvement of the upgraded model based on the few-shot learning paradigm.

Additionally, Table 3 shows the performance of our models on the STARE dataset. Apparently the Impro model generally obtains better results than the Base model, especially in the accuracy, specificity, and AUC metrics. Compared with other methods, our Impro model performs well in sensitivity and achieves 83.37%, which shows that the model possesses good capability to segment vessel pixels. Nevertheless, this may make the upgraded model recognize the more true negatives as true positives and result in poor performance on other metrics, which encourages us to continue to optimize the few-shot based method for vessel segmentation comprehensively. In addition, we can find that the data-driven models are still dominant in these four metrics overall. This is because more images can make this kind of vessel segmentation model, learn the characteristics of blood vessels more fully, and enhance its sensitivity to the information of blood vessels.

**Table 3:** Performance comparison on STARE.

**Methods**		**Acc (%)**		**Spe (%)**		**Sen (%)**		**AUC (%)**	
		**Base**	**Impro**	**Base**	**Impro**	**Base**	**Impro**	**Base**	**Impro**
Proposed	5-way 3-shot	94.59	95.63	96.71	97.39	77.70	81.53	95.63	96.92
	4-way 3-shot	94.46	**95.92**	96.50	98.16	78.18	78.04	95.91	97.29
	3-way 3-shot	94.89	95.81	97.06	97.73	77.56	80.47	96.06	97.16
	3-way 4-shot	94.88	95.77	97.33	97.69	75.39	80.46	95.97	97.25
	3-way 5-shot	94.63	95.65	96.64	97.19	78.51	**83.37**	96.19	**97.53**
Machine learning	Fraz et al. (14)	95.34		97.63		75.48		97.68	
	Aslani and Sarnel (16)	96.05		98.37		75.56		97.89	
	Orlando et al. (17)	-		97.38		76.80		-	
	Li et al. (18)	96.28		98.44		77.26		98.79	
	Srinidhi et al. (19)	95.02		97.46		83.25		96.70	
	Liskowski and Krawiec (22)	97.29		98.62		**85.54**		**99.28**	
	Mo and Zhang (23)	96.74		98.44		81.47		98.85	
	Jiang et al. (24)	97.34		98.46		83.52		99.00	
	Zhou et al. (25)	95.85		97.61		80.65		-	
	Yan et al. (26)	96.12		98.46		75.81		98.01	
	Filipe et al. (27)	96.94		98.58		83.15		99.05	
	Park et al. (28)	**98.76**		**99.38**		83.24		98.73	

*The bold values indicate the maximum value of the corresponding metric in the manuscript*.

Moreover, the two models also performed well on the CHASEDB dataset, as shown in Table 4. The performance of the Impro model is still outstanding, and most of its segmentation evaluation metrics are beyond the Base model under different *CK* modes. The optimal accuracy, sensitivity, specificity, and AUC values achieved by the Impro model are 96.96, 83.44, 98.05, and 97.85%, respectively, which are inferior to those in (24, 27, 28). The reason is that the methods in (24) and (27) both utilized the data augmentation strategy that makes the deep learning framework obtain a more powerful ability to capture the vessel information, and the adversarial learning strategy promotes the scheme (28) to gain strong data distribution learning ability in the vessel segmentation task. Nevertheless, the above accuracy, sensitivity, and specificity values achieved by the Impro model are generally higher than those acquired by the other listed methods, and even the lowest sensitivity value (i.e., 79.08%) obtained from the Base model shows its advantages, which demonstrates the competitiveness of the two models to a certain extent.

**Table 4:** Performance comparison on CHASEDB.

**Methods**		**Acc (%)**		**Spe (%)**		**Sen (%)**		**AUC (%)**	
		**Base**	**Impro**	**Base**	**Impro**	**Base**	**Impro**	**Base**	**Impro**
Proposed	5-way 3-shot	96.76	96.91	97.83	97.92	80.82	81.86	96.64	97.10
	4-way 3-shot	96.85	96.96	97.97	97.99	80.14	81.60	96.92	97.12
	3-way 3-shot	96.85	96.91	98.05	97.87	79.08	82.60	97.48	**97.85**
	3-way 4-shot	96.90	**96.96**	**98.05**	98.02	79.63	81.18	96.93	97.07
	3-way 5-shot	96.70	96.82	97.70	97.72	81.83	**83.44**	97.12	97.24
Machine learning	Fraz et al. (14)	94.69		97.11		72.24		97.12	
	Orlando et al. (17)	-		97.12		72.77		95.24	
	Li et al. (18)	95.81		97.93		75.07		97.16	
	Mo and Zhang (23)	95.99		98.16		76.61		98.12	
	Jiang et al. (24)	96.68		97.45		**86.40**		98.10	
	Zhou et al. (25)	95.20		97.51		75.53		-	
	Yan et al. (26)	96.10		98.09		76.33		97.81	
	Filipe et al. (27)	96.53		**98.64**		77.79		98.55	
	Park et al. (28)	**97.36**		-		-		**98.59**	

*The bold values indicate the maximum value of the corresponding metric in the manuscript*.

### Comparison of the Domain Adaptability of Single Model

**Figure 10:**
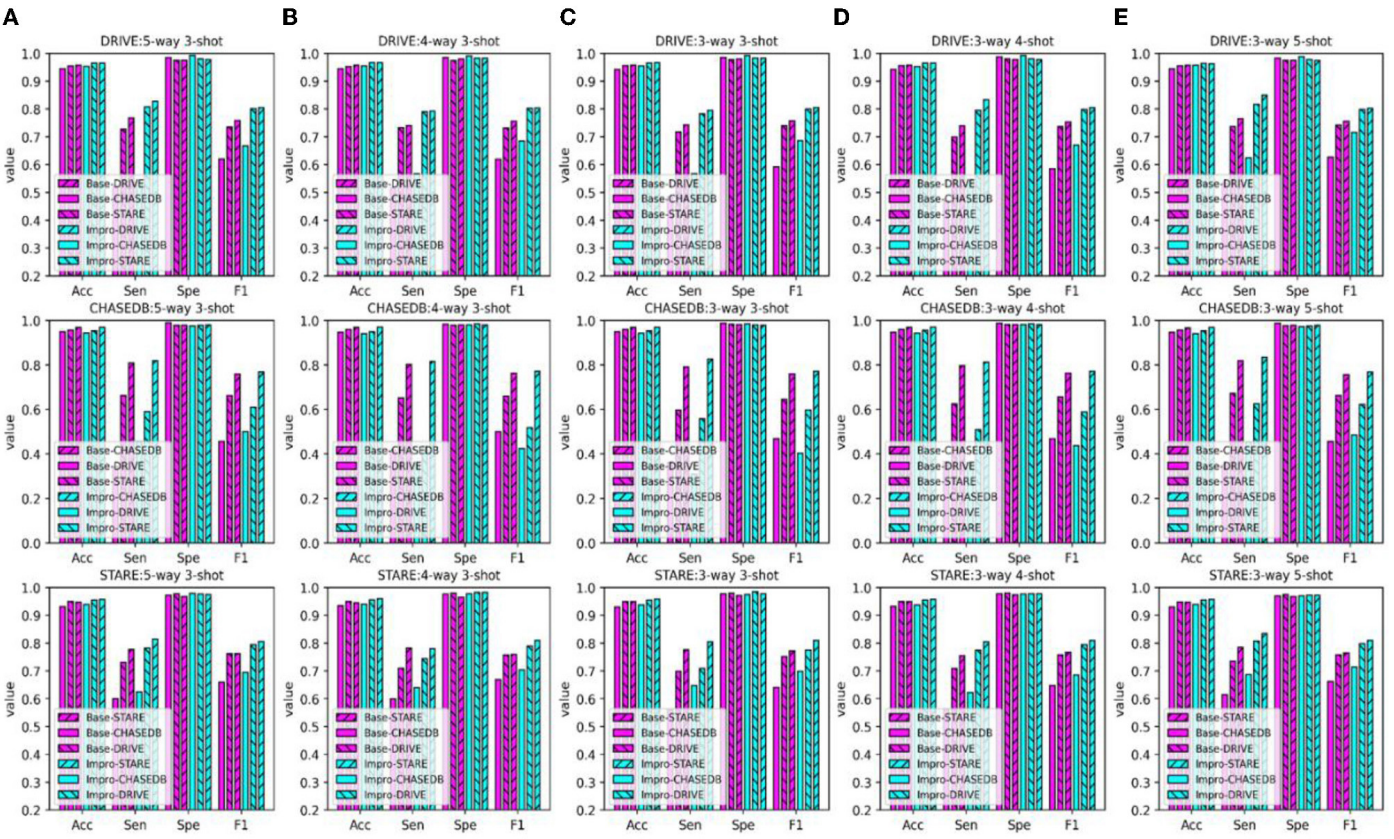
Comparison of the domain adaptability based on the Acc, Sen, Spe, and F1: **(A)** 5-way 3-shot; **(B)** 4-way 3-shot; **(C)** 3-way 3-shot; **(D)** 3-way 4-shot; and **(E)** 3-way 5-shot.

**Figure 11:**
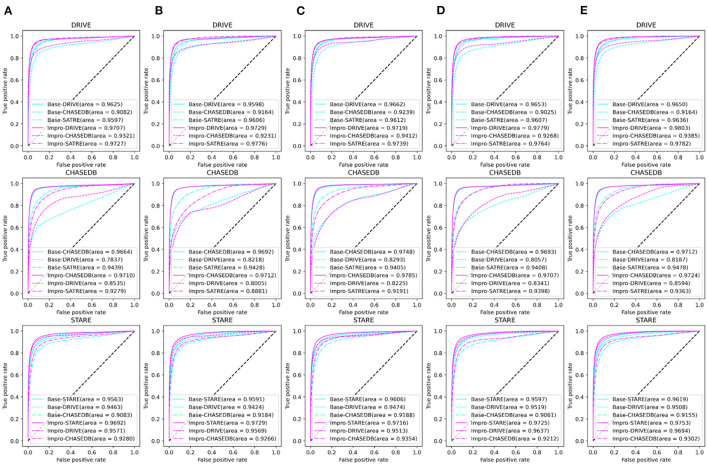
Comparison of the domain adaptability based on the AUC: **(A)** 5-way 3-shot; **(B)** 4-way 3-shot; **(C)** 3-way 3-shot; **(D)** 3-way 4-shot; and **(E)** 3-way 5-shot.

The performance of the DL model in the intra- and cross-domain tasks is the key reference and an important basis to observe its generalization level. Therefore, the intra- and cross-domain vessel segmentation based on a single model (i.e., the baseline model or upgraded model) is performed in this part, and the results are shown in Figures 10, 11. Here, we give an example to illustrate the experimental setup. When experiments are conducted on the STARE dataset, the Base-STARE or Impro-STARE represents an intra-domain experiment, which means that the training set of STARE is used to train the model, and the STARE testing set is used to test the model. On the premise of STARE dataset, the Base-DRIVE or Impro-DRIVE denotes a cross-domain experiment, which represents training the model with a training set from the DRIVE dataset and testing the model based on the STARE testing set.

As shown in Figure 10, the baseline model or the upgraded model generally performs better in the intra-domain task, especially the sensitivity and f1-score metrics. As for the cross-domain scene, the above metric values of the two models are inferior to those in the intra-domain scene. However, as for the other two metrics, it can be found that the performance of the two models in the cross-domain task is close to or even higher than that in the intra-domain task. In addition, it can be seen from the AUC metric in Figure 11 that the retinal vessel segmentation of the two models in the intra-domain scene is superior to that in the cross-domain task in most cases. However, in some instances, the cross-domain segmentation results of the Impro model are even better. For example, when the upgraded model trained based on the STARE dataset was tested on the DRIVE dataset, the AUC values of 97.27, 97.76, and 97.39% were obtained under the conditions of 5-way 3-shot, 4-way 3-shot, and 3-way 3-shot modes respectively, which were 0.2, 0.47, and 0.2% more than the intra-domain values of the corresponding upgraded model. Yet, with the same *CK* modes, the baseline model performs slightly worse in AUC value. The above observations show that the adaptability of a single model in intra- and cross-domain tasks is different, and overall, the intra-domain segmentation level of the model is superior to its cross-domain performance. Nevertheless, in some cases, the good results of the model in cross-domain task also suggest its potential application value in this scenario.

### Comparison of the Domain Adaptability Between the Single Model and the Integrated Framework

The above experiments tested the performance of the baseline and the upgraded models on the public retinal image datasets, which not only showed the effectiveness of the two models in the fundus vessel segmentation task, but also implied their potential competitiveness compared with the existing methods. Of note, whether it is the baseline model or the upgraded model, their performance in the intra-domain task is relatively better than that in the cross-domain task to a certain extent. However, we are also pleased to observe that the cross-domain adaptability of the upgraded model is, sometimes, even more prominent than that of the intra-domain model.

**Figure 12:**
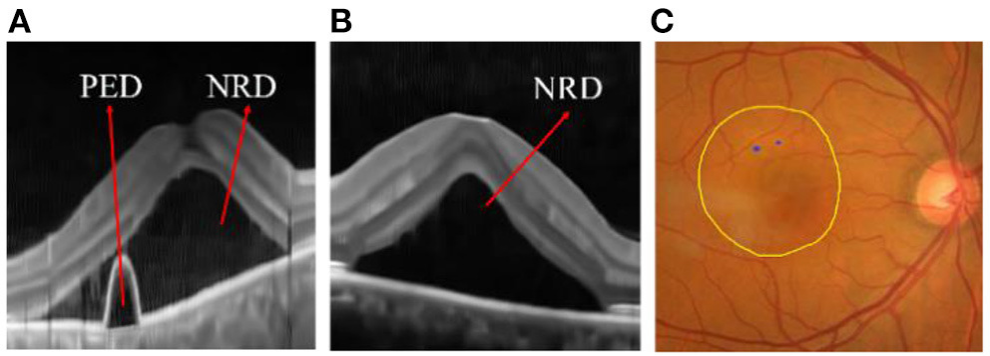
**(A)** Pigment epithelium detachment (PED) and neurosensory retinal detachment NRD; **(B)** NRD **(C)** detachment area (DA) (the internal area marked by yellow line) and leakage point (LP) (the blue point).

In order to improve the cross-domain adaptability of the two models, we proposed an integrated framework (as shown in Figure 4) and verified it on the ophthalmic clinical CSC dataset. The CSC is a common fundus disease caused by the impairment of retinal pigment epithelium function due to the increased choroidal permeability, characterized by neurosensory retinal detachment (NRD), with or without pigment epithelium detachment (PED) (45–47), or detachment area (DA), and leakage point (LP). As shown in Figure 12, the NRD and PED can be clearly displayed on the optical coherence tomography (OCT) B-scan image and DA, and the LP can also be drawn on a fundus color image by an ophthalmologist according to the corresponding angiography image. In recent years, either the traditional laser photocoagulation or the micro-pulse laser photocoagulation has become one of the important means of CSC therapy, which plays an effective role in inhibiting the development of CSC. However, the location of retinal vessels is of great importance in the whole process of the above CSC laser surgery, which does not only help the doctors mark the location of the LP on fundus color image, but also avoids the damage of laser spot to vessel tissue and the low efficiency of surgery caused by the absorption of laser energy by retinal vessels.

Therefore, this section applies the proposed single model (i.e., the baseline model and upgraded model) and the integrated framework to the ophthalmic clinical CSC dataset, which is not only a comparison of their performance, but also a practical application test. First, the symbols in the following figures and tables are briefly explained here. The “Base-DRIVE,” “Base-CHASEDB,” and “Base-STARE” denote the baseline model trained with DRIVE, CHASEDB, and STARE datasets, respectively. the “Base-UNION” represents the integration of the above three models, which means that *N*_1_ is 3. The naming rules for the upgraded models are consistent with the baseline models.

**Figure 13:**
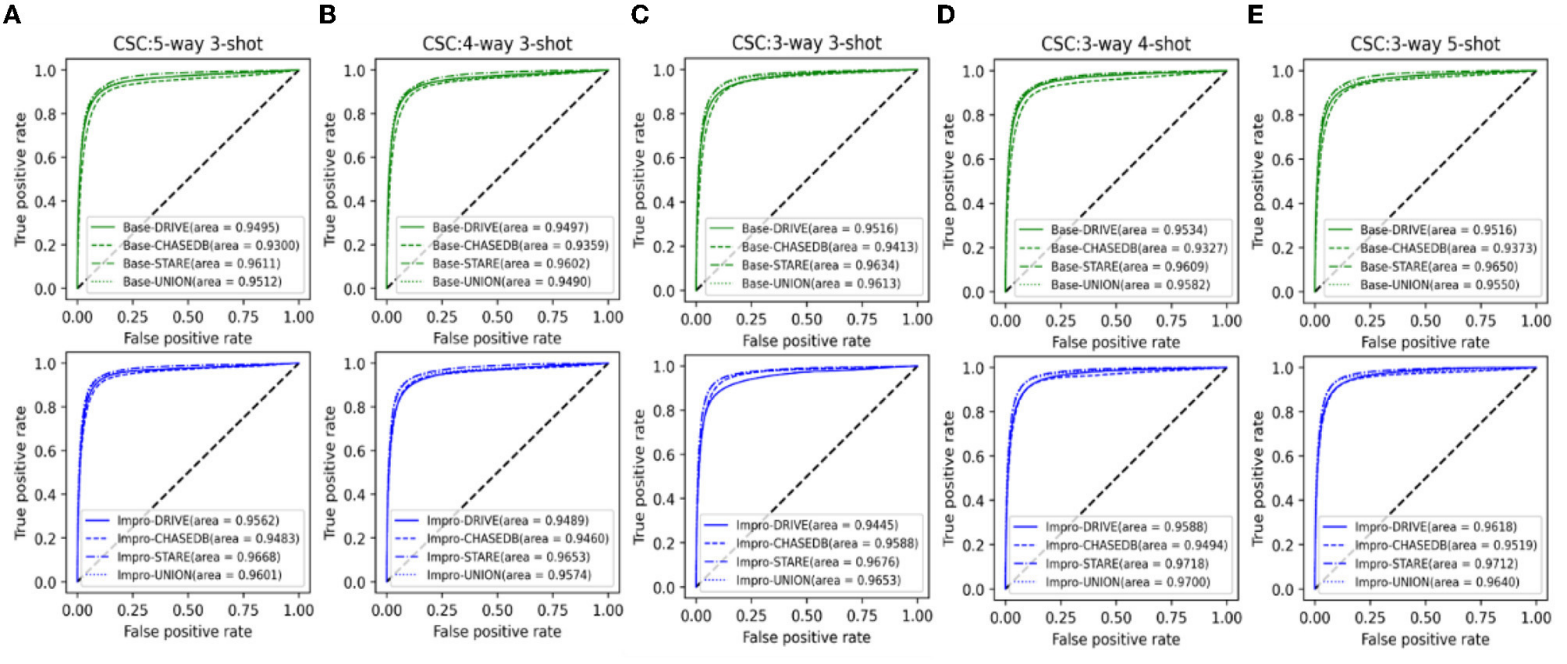
Comparison of the domain adaptability based on the AUC: **(A)** 5-way 3-shot; **(B)** 4-way 3-shot; **(C)** 3-way 3-shot; **(D)** 3-way 4-shot; and **(E)** 3-way 5-shot.

**Figure 14:**
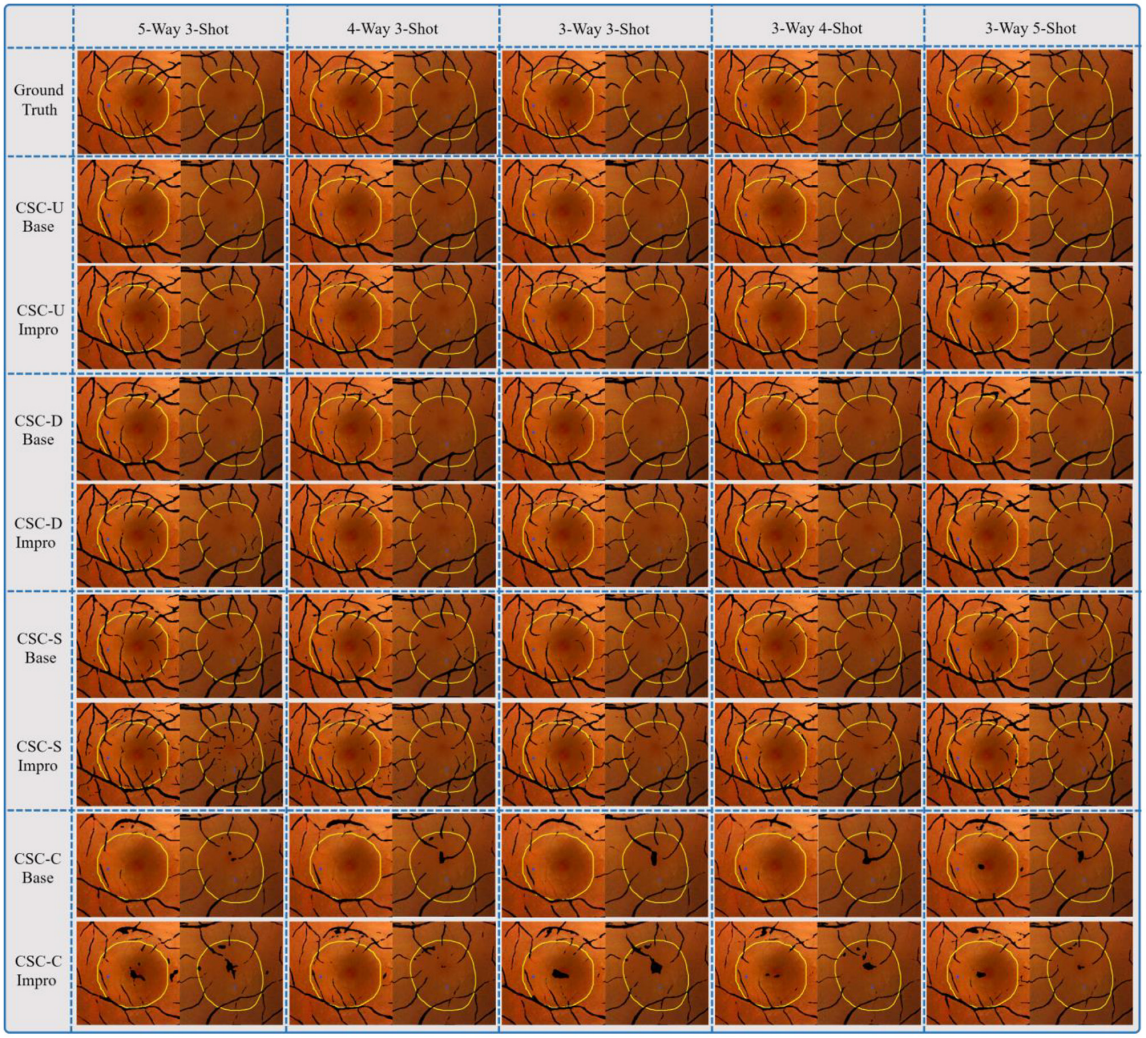
Comparison of the single model and the integrated framework on practical application.

It can be seen from Figure 13 that both the baseline and the upgraded models can better adapt to the CSC dataset, and more than 90% of the AUC values are obtained. In most cases, the AUC metric of the Base-UNION is better than the Base-DRIVE and the Base-CHASEDB; the maximum values of which are 96.13 and 97% under the conditions of baseline and upgrade schemes, respectively. This preliminarily shows the effectiveness and the feasibility of the integrated idea in improving the cross-domain adaptability of a single model. However, we have to admit that the AUC values of the Base-STARE and the Impro-STARE are slightly better than the corresponding integrated models, the maximum values of which are 96.5 and 97.18%, respectively. The above observations, on the one hand, show the correctness of the integration scheme, and on the other hand, also convey that the performance of this scheme has room for further improvement. At the same time, it can also be found that whether the integration framework is based on the single upgraded model or the single upgraded model itself, their AUC values on the CSC dataset is better than their corresponding baseline model in most cases, which is basically consistent with the performance of the upgraded model and the baseline model on the public datasets. The above analysis not only confirms further the necessity of our upgraded operation, but also hints the potential application ability of the model based on the few-shot learning paradigm in clinical dataset. In addition, Table 5 clearly shows the values of the other four metrics of the single model and the integrated framework on the CSC dataset. For example, the CSC-U (U represents Union, that is, the integrated framework) represents the application of the integrated framework on the CSC dataset, and CSC-D represents the application of Base-DRIVE or Impro-DRIVE on the CSC dataset. Under different *CK* modes, the ACC and F1 values of the integrated framework are almost better than those of the corresponding single model, especially the F1 value. Although the integrated framework is slightly inferior in the Spe and Sen metrics, it still has the upper hand overall. It can be found that both the Base-CHASEDB and the Impro-CHASEDB achieved the maximum Spe value, but performed poorly in the Sen metric, which implies that they will introduce more false positives (refer to Figure 14 for details) in vessel segmentation task on the CSC dataset. The above implies that a simple model integration can appropriately enhance the poor cross-domain adaptability of a single model, but the integrated principle of the minority obeying the majority needs to be improved.

**Table 5:** Performance comparison of single model and integrated framework on CSC dataset.

**Methods**		**Acc (%)**		**Spe (%)**		**Sen (%)**		**F1 (%)**	
		**Base**	**Impro**	**Base**	**Impro**	**Base**	**Impro**	**Base**	**Impro**
5-way 3-shot	CSC-U	**94.90**	**95.09**	96.32	96.08	79.40	84.25	**72.30**	**74.20**
	CSC-D	94.52	94.46	96.08	95.56	77.48	82.45	70.33	71.38
	CSC-S	93.93	94.19	94.91	94.83	**83.23**	**87.21**	69.68	71.55
	CSC-C	94.06	94.82	**96.57**	**96.96**	66.63	71.45	65.30	69.83
4-way 3-shot	CSC-U	**95.01**	**95.32**	96.47	96.58	79.07	81.53	**72.65**	**74.48**
	CSC-D	94.74	94.57	96.39	96.09	76.67	77.98	70.96	70.67
	CSC-S	93.83	94.84	94.79	95.90	**83.31**	**83.30**	69.35	73.03
	CSC-C	94.26	94.87	**96.77**	**96.94**	66.90	72.28	66.17	70.28
3-way 3-shot	CSC-U	**95.07**	**95.19**	96.62	96.34	78.09	82.63	**72.64**	**74.24**
	CSC-D	94.60	94.49	96.39	96.07	74.98	77.23	69.94	70.16
	CSC-S	94.29	94.54	95.37	95.31	**82.47**	**86.06**	70.78	72.53
	CSC-C	94.12	94.77	**96.91**	**96.67**	63.61	73.93	64.45	70.31
3-way 4-shot	CSC-U	**95.18**	**95.06**	96.76	96.10	77.82	83.72	**73.01**	**73.97**
	CSC-D	94.72	94.27	96.43	95.49	76.05	80.84	70.73	70.27
	CSC-S	94.43	94.43	95.66	95.17	**81.02**	**86.35**	70.93	72.22
	CSC-C	94.23	94.83	**97.03**	**97.05**	63.53	70.56	64.85	69.60
3-way 5-shot	CSC-U	**94.85**	94.67	96.17	95.45	80.33	86.19	**72.33**	**73.06**
	CSC-D	94.36	93.93	95.93	94.86	77.21	83.68	69.66	69.79
	CSC-S	93.92	93.77	94.79	94.23	**84.40**	**88.78**	69.95	70.50
	CSC-C	94.07	**94.78**	**96.44**	**96.56**	68.16	75.35	65.84	70.77

*The bold values indicate the maximum value of the corresponding metric in the manuscript*.

Figure 14 shows the fusion results of the segmented retinal vessels and the fundus color images, in which the black part represents the position information of vessels, the inner area marked by yellow line represents DA, and the blue point is LP. After a careful comparison, it can be found that the integrated framework can segment more retinal vessels than the single model driven by DRIVE or CHASEDB dataset. Although the Base-CHASEDB and the Impro-CHASEDB have advantages in Spe metric, it leads to more false positives (Refer to Table 5) in the actual segmentation results. Moreover, the Base-STARE or the Impro-STARE is generally dominant in the task of vessel segmentation. However, it cannot be ignored that they will bring more discrete vessel segments, which may disturb the LP localization process.

In general, through the above results and discussions, it can be found that the performance of both the Base and Impro models is consistent in the training, validation, and testing processes, which indicates the stability of the two models. In addition, we can see that although the baseline model can adapt to the vessel segmentation task well, it is weaker than the Impro model in terms of adhesion and continuity in wide vessel segmentation as a whole, which also emphasizes the necessity of an upgraded operation. Moreover, compared with some typical machine-learning-based methods listed, the Base and Impro models show superiorities in most cases, but we still need to catch up with the optimal scheme. Furthermore, a single model performs well in the intra- and cross-domain tasks, and overall, either the baseline model or the upgrade model is better in intra-domain tasks. The proposed integrated framework enhanced the vessel segmentation ability of a single model in the cross-domain task to a certain extent, but the integrated idea based on the minority obeying majority needs to be improved in the future to promote the superiority of the integrated framework.

## Conclusion and Future Work

In this paper, a novel few-shot learning-based method for retinal vessel segmentation is proposed. Firstly, from the perspective of a problem scenario migration, the vessel segmentation scene is skillfully transformed into the few-shot learning task, which lays a foundation for vessel segmentation under the condition of few samples. Then, on the basis of the above, two models based on the few-shot learning paradigm are established. In the first step, we build a baseline segmentation model by improving the existing few-shot learning framework, which adapts to the vessel segmentation task well. In the second step, to improve the segmentation performance of the baseline model, we make further efforts from the following three aspects: (1) A multi-scale class prototype extraction technique is designed to obtain more sufficient vessel features for better utilizing the information from the support images; (2) The multi-scale vessel features of the query images inferred by the support image class prototype information are gradually fused to provide more effective guidance for the retinal vessel segmentation task; and (3) A multi-scale attention module is raised to promote the consideration of the global information in the upgraded model to assist the vessel localization. Moreover, the integrated framework is conceived to appropriately alleviate the low performance of a single model in the cross-domain vessel segmentation scene. Extensive experiments on three public retinal image datasets demonstrate that the few-shot learning-based method can effectively carry out the intra-domain vessel segmentation task with a few annotation samples and possess a potential cross-domain application capability. Practical application experiments on our private CSC dataset not only confirms the effectiveness of the integrated framework to improve the cross-domain adaptability of a single model, but also further indicates the clinical application value of the few-shot learning-based method in assisting the CSC laser surgery for retinal vessel localization. However, although we broaden the research ideas for the vessel segmentation in the case of few samples, the limitations of this method cannot be ignored. In the future, we will focus on enhancing the segmentation ability of the proposed models on the small vessels and alleviate the problem of a discontinuous segmentation issue, and try to explore the vessel segmentation scheme based on the few-shot learning paradigm without any annotations.

## Data Availability Statement

The public fundus datasets (i.e., DRIVE, STARE, and CHASEDB datasets) for this study can be found in the corresponding references. The private clinical fundus CSC dataset used and analyzed in our research are available from the corresponding author upon request.

## Ethics Statement

The studies involving human participants were reviewed and approved by Ethics Committee of the Affiliated Eye Hospital of Nanjing Medical University. The patients/participants provided their written informed consent to participate in this study.

## Author Contributions

JX developed the presented method and drafted the manuscript. JS and WY supervised and reviewed the whole project. CW assisted in improving the manuscript. QJ and ZY provided suggestions for the clinical application of the study and helped to analyze the experimental results. All authors contributed to the article and approved the submitted version.

## Funding

This work was financially supported by Fundamental Research Funds for the Central Universities (No. NP2020420), China Postdoctoral Science Foundation Funded Project (No. 2019M661832), and the Nanjing Enterprise Expert Team Project.

## Conflict of Interest

The authors declare that the research was conducted in the absence of any commercial or financial relationships that could be construed as a potential conflict of interest.

## Publisher's Note

All claims expressed in this article are solely those of the authors and do not necessarily represent those of their affiliated organizations, or those of the publisher, the editors and the reviewers. Any product that may be evaluated in this article, or claim that may be made by its manufacturer, is not guaranteed or endorsed by the publisher.
